# The Effectiveness of GLP-1 Receptor Agonist Semaglutide on Body Composition in Elderly Obese Diabetic Patients: A Pilot Study

**DOI:** 10.3390/medicines9090047

**Published:** 2022-09-16

**Authors:** Yoshinori Ozeki, Takayuki Masaki, Akari Kamata, Shotaro Miyamoto, Yuichi Yoshida, Mitsuhiro Okamoto, Koro Gotoh, Hirotaka Shibata

**Affiliations:** 1 Department of Endocrinology, Metabolism, Rheumatology and Nephrology, Faculty of Medicine, Oita University, Yufu City 879-5593, Oita, Japan; 2 Faculty of Medicine, Oita University, Yufu City 879-5593, Oita, Japan

**Keywords:** GLP-1 receptor agonist, semaglutide, obesity, diabetes, body composition

## Abstract

Background and Objectives: This study aimed to investigate the changes in obesity severity, glucose metabolism, and body composition in patients with obesity and type 2 diabetes mellitus treated with glucagon-like peptide 1 receptor agonist (GLP1-RA) semaglutide. Materials and Methods: Body weight (BW), metabolic parameters, and body composition were examined before and 3 months after semaglutide administration. The mass of body fat (FM), fat weight percentage (%FM), mass of skeletal muscle (MM), skeletal MM percentage (%MM), and limb muscles were measured using the bioelectrical impedance method. Results: Semaglutide dramatically reduced the weight, the body mass index (BMI), and the levels of the glucose metabolic markers, including fasting blood glucose and hemoglobin A1c, and accelerated the loss of excess BW. FM, MM, and %FM after semaglutide treatment also decreased. Conversely, semaglutide had no effect on the %MM after 3 months. In limb muscle analyses, right upper and lower leg muscle percentages, left upper and lower leg muscles, and the ratios of the lower/upper muscles were maintained by semaglutide treatment. Conclusions: These results suggest that the GLP1-RA semaglutide effectively reduces body adiposity while maintaining the MM in obese type 2 diabetic patients.

## 1. Introduction

Obesity is one of the risk factors for several metabolic diseases [1,2]. In patients with obesity, body weight (BW), mass of body fat (FM), and mass of skeletal muscle (MM) are related to type 2 diabetes mellitus (T2DM) [3,4]. Previous reports have demonstrated body composition is involved in glycemic control and relative changes in fat weight and lean body mass (LBM) during T2DM treatment [5,6]. In patients with obesity, LBM usually decreases with the body fat reduction that accompanies total BW (TBW) loss [7]. However, a decrease in MM may worsen the glycemic control of diabetic patients [8,9]. Decreased MM is also related to increased risks of sarcopenia and frailty [10,11]. Thus, during BW loss, a decrease in FM without a significant loss of MM is preferable.

Several techniques for the assessment of MM have been evaluated [12,13]. These include bioelectrical impedance methods (BIA), which are simple methods for assessing body fat and muscle [14,15,16] based on differences in electrical movements through body water and several tissues [15,16,17]. Body composition measurements divide the body into areas based on differing tissue properties. These compartments include FM, skeletal MM, total body water, and bone mineral content [18]. Thus, BIA can be used to assess body composition in obese diabetic patients [18].

The medications used to treat patients with obesity and T2DM include glucagon-like peptide 1 receptor agonists (GLP1-RAs) and sodium-glucose co-transporter 2 inhibitors (SGLT2is), both of which have additional clinical benefits beyond improving glucose homeostasis and promoting weight loss [19]. SGLT2is cause the proximal renal tubule to inhibit glucose reabsorption [20]. In patients with obesity and T2DM, treatment with SGLT2is decreases FM and LBM [21,22]. Studies have suggested that SGLT2is have catabolic effects on FM and MM through an increase in glycosuria. GLP1-RAs delay gastric emptying and induce satiety, leading to decreases in BW and FM [23]. They also exert multiple effects on LBM [24,25,26,27,28]. A stepwise increase in the contribution of LBM was described in patients prescribed the GLP1-RA liraglutide in the LEAD-2 trial [24]. However, a smaller, or even no, influence on LBM with regard to the body weight loss induced by liraglutide treatment of patients with obesity and T2DM has also been described [25,26,27,28].

Semaglutide is a new GLP1-RA with high homology to human GLP-1. It has a long half-life and can be injected once weekly to induce strong weight-loss effects [29]. However, the change in body composition associated with the weight loss induced by semaglutide, especially the change in MM, is unclear. Therefore, in this study, we examined body composition, particularly skeletal MM, in Japanese patients with obesity and T2DM treated with semaglutide.

## 2. Materials and Methods

### 2.1. Patients

Forty-eight patients with obesity and T2DM undergoing semaglutide treatment at Oita University Hospital from August 2020 to June 2021 were retrospectively recruited for this study. Patients were administered semaglutide therapy in accordance with the standardized inclusion criteria of Japan. The patients had heart disease as determined by chest X-ray and echocardiography, and patients with endocrine diseases were excluded. Twenty-two patients with drop-off and medical treatments were changed during the study of 48 screened patients. Thirteen patients could not perform the in-body composition examination at an appropriate time. Thus, 13 fulfilled all the very narrow inclusion criteria and were eligible for study entry. Eleven patients were taking anti-diabetic drugs before or during the study (sodium-glucose transport protein 2 inhibitors, eight patients; metformin, four patients; dipeptidyl peptidase-4 inhibitor, three patients; sulfonylureas, one patient; and others, two patients). The present study design was based on the Declaration of Helsinki and was permitted by the Ethical Committee of Oita University (13 July 2018 as protocol code 762).

### 2.2. Blood Sampling

Blood was collected at 8:00–11:00 A.M. from the vein in overnight-fasted patients. All patients were assessed according to their levels of fasting blood glucose (FPG), hemoglobin A1c (HbA1C), alanine transaminase (ALT), aspartate transaminase (AST), gamma-glutamyl transpeptidase, triglycerides, high-density lipoprotein (HDL), low-density lipoprotein (LDL), creatinine (Cr), and blood urea nitrogen (BUN). 

### 2.3. Bioelectrical Impedance Analysis

Body composition was assessed by a BIA apparatus (In-Body 770; Bio space Co., Tokyo, Japan), as described in Section 1. The apparatus has five body areas: right and left upper legs, trunk, and right and left lower legs [18]. Body fat evaluated using the BIA apparatus correlated with that measured by dual-energy X-ray absorptiometry [30]. Body composition was examined with the patient in a standing position and calculated using the software provided by the manufacturer. Fat weight, fat weight percentage (%FM), MM, bone content, and body fluid were also assessed. %FM was calculated as FM (kg)/BW (kg) × 100, and MM percentage was calculated as MM (kg)/BW (kg) × 100.

### 2.4. Statistics

All data are presented as the mean ± standard deviation examined by Statistical software (JMP14.1; SAS Institute, Cary, NC, USA). Changes in metabolic parameters were evaluated by the Wilcoxon test. Statistical significance was cut off at *p* < 0.05.

## 3. Results

### 3.1. Basal Clinical Data of Patients and BW Changes after Semaglutide Administration

The data of the patients before semaglutide treatment are shown in Table 1. BW and BMI decreased from their pre-treatment values 3 months after semaglutide administration. 

The average age is 52.0 ± 6.9 years, and the diabetic duration followed by medical treatment or a specific diet is 11.1 ± 11.3 years (Table 1).

### 3.2. Changes in Blood Metabolic Parameters after Semaglutide Administration 

Compared to their pre-treatment values, the FPG and HbA1C levels were significantly lower 3 months after semaglutide treatment. Conversely, there was no significant difference in AST, ALT, GTP, triglycerides, LDL, HDL, BUN, or Cr values throughout the study period (Table 1).

### 3.3. Changes in Fat Mass, Body Fat Percentage, Ratio of Extracellular Fluid, and Bone Mineral Content after Semaglutide Treatment

Data on the changes in FM, %FM, proportion of extracellular fluid, body fluid, and bone mineral content over time are presented in Table 2. FM and %FM were markedly lower at 3 months than prior to treatment (*p* < 0.05), whereas no significant change was observed in bone mineral content throughout the study (*p* > 0.1).

### 3.4. Changes in MM and %MM after Semaglutide Treatment

Data on skeletal MM over time are presented in Table 2. Although skeletal MM decreased, skeletal MM percentage after 3 months of semaglutide treatment was maintained. The changes in MM of the upper and lower extremities after semaglutide are presented in Table 3. There were no changes in the upper and lower leg MM percentages or in the ratio of lower/upper MM after 3 months of semaglutide treatment.

## 4. Discussion

In the present study, 3 months of semaglutide treatment dramatically reduced TBW, BMI, blood glucose, and HbA1c levels in obese diabetic patients with obesity and T2DM. Although fat weight and %FM were dramatically reduced, no significant changes were observed in bone mineral content. A previous study also showed that semaglutide improved BMI, FPG, and HbA1c levels [31]. Notably, according to the present study, semaglutide maintained skeletal MM and %MM, particularly upper and lower leg MM percentages and lower/upper MM ratio.

The weight was loss related to body composition, including FM and MM. Obese patients have an increase in fat weight and a body fat increase that affects metabolic diseases [32]. In the present study, the patients exhibited an FM decrease after semaglutide treatment, indicating that loss of fat is important for improving HbA1c levels. Thus, our study suggests that, by inducing FM loss, semaglutide is beneficial in patients with obesity and T2DM.

In contrast to fat loss, a reduction in skeletal muscle is not good for diabetic obese patients. In general, losses in FM and MM occur after caloric restriction and bariatric surgery [18,24,25,26,27,28]. However, studies of the effects of GLP1-RAs on LBM and MM have produced relatively inconsistent findings. In the LEAD-3 trial, liraglutide decreased body weight after treatment and induced loss of LBM [24].

There are also studies reporting no change, or increases, in LBM after liraglutide treatment [27,28]. Similar to liraglutide, the relative contribution of fat weight to body weight loss induced by exenatide ranged from 40% [33] to 52% [34]. Other studies reported a small increase in LBM after exenatide treatment in patients with T2DM [35]. The loss of fat weight was greater than the TBW decrease and was accompanied by an increase in skeletal muscle [36]. Our study also demonstrated that %MM is conserved in subjects treated with semaglutide.

The reasons for the heterogeneous findings include the dose of GLP-RAs, the duration of treatment, the amount of weight loss, the patient’s condition, and concomitant therapies (alongside GLP1-RAs). A recent study showed a mean reduction of LBM of 1.1 kg after 12 weeks of semaglutide treatment, with dose escalation to 1.0 mg [37]. This magnitude of LBM loss is close to that observed in the patients in the present study.

GLP1-RAs include short-acting compounds, such as exenatide, which provide several hours of GLP-1R activation, and long-acting compounds, such as dulaglutide and semaglutide, which activate GLP-1R continuously over a week [29]. The differences in the pharmacokinetics of these drugs in patients of different races may lead to differences in their effects on body composition.

We described the association between body composition and glucose homeostasis in obese diabetic patients after bariatric surgery in a previous study [18]. The postoperative muscle weight was related to the blood glucose and HbA1c levels. Although the interventions were different, the results of this and our previous study suggest that sustained MM in patients with obesity and T2DM after semaglutide treatment is closely related to FPG and HbA1c levels.

GLP-1 exerts an antidiabetic effect through multiple mechanisms, such as reduced food intake, gastric emptying, decreased FM, and skeletal MM conservation [35,36]. We recently reported that semaglutide improves eating habits, and the sensation of hunger is correlated with glucose homeostasis [38]. In this study, although food intake and gastric emptying were not assessed, skeletal MM was preserved after semaglutide treatment. The ratios of U/W, L/W, and U/L were maintained compared to their pre-treatment values. GLP1-RAs activate glucose delivery in skeletal muscle through an AMP-activated protein kinase (AMPK) [39,40].

GLP-1 also mediates increases in vascular blood flow in muscles, suggesting that it increases the glucose transport to the tissue, thus increasing muscle synthesis and decreasing muscle breakdown [41]. An enhancement of skeletal MM induced by GLP1-RA offers benefits in terms of glucose regulation. Moreover, enhanced MM has positive effects on insulin resistance and fat metabolism through several molecular mechanisms [42]. Therefore, strategies to preserve MM and physical muscle function in patients with obesity, through structured exercise, can play an important role in achieving normal glucose levels. Notably, muscular strength and flexibility are important, along with absolute skeletal MM [43]. In previous studies, semaglutide was shown to have strong anti-diabetic effects compared to other types of similar medications [31,44,45]. It is possible that semaglutide effectively increases vascular blood flow in muscles, suggesting that it promotes muscle synthesis. Further studies are required to validate the effects of semaglutide on these muscle parameters.

SGLT2is were shown to decrease LBM in patients with obesity and T2DM [21,22], whereas in studies of GLP1-RA, there were no changes, or increases, in LBM after treatment [27,28]. These results suggest that a combination of GLP1-RAs and SGLT2is will prevent LBM loss during dieting. SGLT2is increase, while GLP1-RAs decrease, food intake in humans and rodents [46]. A combination of these agents may have additional clinical benefits for hyperphagia. This was indicated by a study in which the administration of GLP1-RAs decreased SGLT2i-induced hyperphagia via the brain AMPK pathway [47].

## 5. Limitations

One limitation of our study is its small sample size. Moreover, the 3-month treatment with semaglutide resulted in very limited results regarding the adiposity and lipid profiles. The MM in kilograms was reduced, but the percentage did not change after short-term semaglutide treatment. Thus, longer and larger prospective studies are required to validate some of the outcomes. 

## Figures and Tables

**Table 1:** Changes in BW and blood metabolic parameters after semaglutide treatment.

	Baseline	3 Months	*p*
Age (years)	52.0 ± 6.9		
Diabetic duration (years)	11.1 ± 11.3		
Body weight (kg)	93.9 ± 14.6	90.8 ± 14.6	<0.01
Total body weight loss (kg)		3.1 ± 2.5	
%TBWL		3.3 ± 2.5	
%EBWL		9.8 ± 7.3	
BMI (kg/m^2^)	35.9 ± 6.1	34.7 ± 5.8	<0.01
Fasting plasma glucose (mg/dL)	116.3 ± 29.8	104.5 ± 36.1	0.03
HbA1c (%)	7.0 ± 1.0	6.4 ± 1.0	<0.01
Triglycerides (mg/dL)	151.0 ± 62.7	152.1 ± 60.3	0.75
HDL cholesterol (mg/dL)	58.9 ± 9.8	57.5 ± 9.6	0.39
LDL cholesterol (mg/dL)	122.1 ± 24.2	109.1 ± 21.7	0.05
BUN (mg/dL)	14.1 ± 3.4	14.5 ± 5.4	0.77
Creatinine (mg/dL)	0.7 ± 0.3	0.8 ± 0.2	0.17
AST (IU/L)	20.3 ± 10.3	21.2 ± 9.4	0.15
ALT (IU/L)	24.1 ± 14.1	25.5 ± 14.0	0.51
GTP (IU/L)	22.5 ± 10.1	22.0 ± 12.7	0.38

%TBWL: percent total body weight loss, %EBWL: percent excess body weight loss, BMI: body mass index, LDL: low-density lipoprotein, HDL: high-density lipoprotein, AST: aspartate aminotransferase, ALT: alanine transaminase, GTP: glutamic pyruvic transaminase, BUN: blood urea nitrogen.

**Table 2:** Time-course changes in body composition after semaglutide treatment.

	Baseline	3 Months	*p*
FM (kg)	40.5 ± 12.7	38.2 ± 12.8	<0.01
FM (%)	42.5 ± 9.5	41.4 ± 10.2	<0.01
Skeletal MM (kg)	29.5 ± 5.3	29.0 ± 5.4	<0.05
Skeletal MM (%)	31.9 ± 5.7	32.3 ± 6.1	0.06
Bone mineral content	2.90 ± 0.63	2.91 ± 0.64	0.76
Ratio of extracellular fluid	0.39 ± 0.01	0.39 ± 0.01	0.79

FM: weight of fat mass, MM: weight of muscle mass.

**Table 3:** Changes in upper and lower leg muscle after semaglutide treatment.

	Baseline	3 Months	*p*
Right upper leg muscle (%)	3.2 ± 0.6	3.3 ± 0.7	0.07
Right lower leg muscle (%)	9.0 ± 1.4	9.0 ± 1.6	0.81
Left upper leg muscle (%)	3.3 ± 0.6	3.3 ± 0.7	0.31
Left lower leg muscle (%)	8.9 ± 1.5	8.9 ± 1.6	0.75
Upper leg muscle (%)	6.5 ± 1.2	6.6 ± 1.3	0.15
Lower leg muscle (%)	18.0 ± 2.9	18.0 ± 3.2	0.81
Lower/Upper leg muscle	2.8 ± 0.2	2.7 ± 0.3	0.10

## Data Availability

The data presented in this study are available upon reasonable request from the corresponding author. The data are not publicly available to preserve patient confidentiality and privacy.
